# Systemic Capillary Leak Syndrome (Clarkson Syndrome) in Cancer Patients: A Systematic Review

**DOI:** 10.3390/jcm7110418

**Published:** 2018-11-06

**Authors:** Jae Il Shin, Keum Hwa Lee, I. Re Lee, Ji Hyun Oh, Dong Wook Kim, Jae Won Shin, Tae Seong Eo, Andreas Kronbichler, Michael Eisenhut, Hans J. van der Vliet

**Affiliations:** 1 Department of Pediatrics, Yonsei University College of Medicine, Yonsei-ro 50, Seodaemun-gu, C.P.O. Box 8044, Seoul 03722, Korea; AZSAGM@yuhs.ac (K.H.L.); jireh18@hanmail.net (I.R.L.); inflames132@naver.com (D.W.K.); aguilera83@naver.com (J.W.S.); djxotjd@gmail.com (T.S.E.); 2 Department of Pediatric Nephrology, Severance Children’s Hospital, Seoul 03722, Korea; 3 Institute of Kidney Disease Research, Yonsei University College of Medicine, Seoul 03722, Korea; 4 Wonkwang University School of Medicine, Iksan 54538, Korea; chamomilez@nate.com; 5 Department of Internal Medicine IV (Nephrology and Hypertension), Medical University Innsbruck, 6020 Innsbruck, Austria; andreas.kronbichler@i-med.ac.at; 6 Luton & Dunstable University Hospital NHS Foundation Trust, Lewsey Road, Luton LU4 ODZ, UK; michael_eisenhut@yahoo.com; 7 Department of Medical Oncology, Amsterdam UMC, Cancer Center Amsterdam, VU University, 1081 HV Amsterdam, The Netherlands; JJ.vanderVliet@vumc.nl

**Keywords:** systemic capillary leak syndrome, cancer, interleukin-2, steroids, intravenous immunoglobulins

## Abstract

Systemic capillary leak syndrome (SCLS) is a rare disease characterized by shock caused by capillary hyperpermeability. The disease can occur in cancer patients and effective therapeutic strategies have not been established yet. The aim of the study was to analyze the clinical and laboratory data, treatment modalities, and mortality rate of patients and to identify contributing factors leading to mortality of SCLS in cancer. We searched MEDLINE (inception to July 2018) and of 4612 articles, we identified 62 case reports on SCLS associated with cancer or cancer-related drugs in a total of 53 articles. SCLS was associated with cancer itself in 43.6%, with anti-cancer agents in 51.6% and bone marrow transplantation (BMT) in 4.8%. Among anti-cancer agents, granulocyte-colony stimulating factor (G-CSF) was the most frequently associated drug (14.6%), followed by interleukin (IL)-2 (11.4%). The most common associated malignancies were hematologic (61.3%) with non-Hodgkin lymphoma (22.7%) and multiple myeloma (12.9%) being the leading causes. Common symptoms and signs included dyspnea (27.4%), edema (67.7%), hypotension (32.2%), pleural effusion (29.0%), ascites (22.7%), oliguria (22.7%), and weight gain (21.0%). Patients with SCLS were treated with steroids (59.7%), volume replacement (33.8%), diuretics (24.2%), inotropes (9.6%), methylxanthines (12.8%), β2 agonists (4.8%), while intravenous immunoglobulins (IVIG) were administered in 2 patients (3.2%) only. Among sixteen deaths during follow-up, four were directly attributed to SCLS. Hematologic malignancies were associated with an increased risk for mortality (hazard ratio (HR) 8.820, 95% confidence interval (CI) 1.126–69.063, *p* = 0.038). Taken together, SCLS can be one important adverse event in cancer patients and careful monitoring of fluid volume is required in the management of SCLS.

## 1. Introduction

Systemic capillary leak syndrome (SCLS), also named vascular leak syndrome (VLS) or Clarkson disease/syndrome, was first reported by Clarkson et al. in 1960 [1]. Clarkson et al. described idiopathic SCLS as a rare disease of reversible plasma extravasation and vascular collapse accompanied by a classic triad of hypoalbuminemia, hemoconcentration and hypotension in the absence of secondary causes of shock [1]. Since then, more than 250 cases of idiopathic SCLS have been reported in the literature and the number of cases increased by more recognition of this disease [2,3,4,5]. SCLS may be idiopathic but can also be caused by other potential factors encountered in a clinical setting, such as drugs, infections, or surgery [4]. Although not fully investigated yet, SCLS can also develop in patients with cancer.

SCLS is a potentially fatal syndrome, since a high mortality rate was reported due to severe shock [2,3,5]. According to the European Clarkson disease (EurêClark) registry data [6], 5- and 10-year survival rates were 78% (*n* = 35) and 69% (*n* = 17), respectively, in the 65 patients with follow-up.

Nevertheless, clinical characteristics, treatments, and outcomes, including mortality rate, have not yet been systemically studied in cancer patients. It is speculated that the non-specific nature of the presenting signs and symptoms of SCLS may have resulted in considerable underdiagnosis despite its high mortality rate. Furthermore, as there are presently no definitive guidelines for the initial and long-term management of SCLS, it is also important to gain more insight into the efficacy of various therapeutic approaches currently used.

In this special situation, many clinicians may not recognize SCLS, which can lead to a delay in the diagnosis and treatment of SCLS, which increases mortality and morbidity. Therefore, there is a need for early and reliable diagnosis of SCLS. The aim of this work was to extend the understanding of SCLS in cancer patients. As no systematic review has been performed investigating the characteristics of cancer patients with SCLS yet, we comprehensively analyzed the clinical and laboratory characteristics, treatment patterns and patient survival in all published cases. Our data may give insight into the clinical course, outcome, and potential therapeutic approaches of cancer-associated SCLS.

## 2. Methods

### 2.1. Literature Search and Study Selection

For this systematic review, we followed the guideline of Preferred Reporting Items for Systematic Reviews and Meta-Analyses (PRISMA) checklist (Supplementary Table S1). We performed a literature search to systematically collect case reports of SCLS associated with cancer or cancer-related drugs. Two investigators (K.H.L. and I.R.L.) independently searched PubMed and EMBASE and performed an extraction of the data. The last search was done on 15th July 2018. The search terms were: “(Capillary leak OR Vascular leak) AND (cancer OR carcinoma OR neoplasm OR tumor)”. We labeled all articles by examining titles, abstracts, full texts in order and any discrepancy was discussed and resolved by consensus between 3 investigators (J.I.S., K.H.L. and I.R.L.).

To determine the eligibility for inclusion in the review, we screened abstracts according to the following criteria: (1) case reports of patients with cancer; and (2) SCLS was attributed to cancer itself or cancer treatment-related drugs. Cases of SCLS which were caused by idiopathic forms, infection, or surgery were excluded from this systematic review. 

Our initial search yielded 4612 articles, but we finally identified 62 case reports in 53 articles that met the inclusion criteria for this systematic review.

### 2.2. Data Extraction

For each eligible case report, we abstracted and recorded information on the name of authors, journal name, publication year/month, age, gender, type of cancer, anti-cancer drugs or agents used, clinical presentations at onset of SCLS, laboratory findings, types of treatment and outcome (alive or death). 

### 2.3. Analyses of Case Reports

We presented the data as frequency for age, gender, type of cancer, anti-cancer agents used, clinical presentation at onset of SCLS, laboratory findings, and types of treatment in tabulated form. The data for each study are presented in Supplementary Table S2. We compared the clinical and laboratory characteristics between being alive or dead during follow-up. 

### 2.4. Statistical Analysis

Statistical analyses were performed, using the SPSS for Windows (SPSS Inc., Chicago, IL, USA) and MedCalc version 15.8 (MedCalc Software, Ostend, Belgium). To identify factors related to mortality in cancer patients with SCLS, various factors (demographic, clinical, laboratory findings, and treatments) were compared between patients who were still alive and those who died. Chi-square or Fisher exact test was performed for the analysis for categorical variables (sex, cause of SCLS, diagnosis of cancer (hematologic or non-hematologic malignancy), presence of hypotension, hypoalbuminemia, leukocytosis and the use of steroid therapy) and the independent *t* test for continuous variables (age, systolic or diastolic blood pressures, serum albumin levels, white blood cell (WBC) or platelet counts, hemoglobin and hematocrit levels). We also performed cox proportional hazard regression to analyze whether one or more covariates might be associated with mortality. Kaplan-Meier analysis using the log rank test was used to test whether the survival of the patients was different according to the clinical, laboratory and treatment characteristics with time. All differences were considered statistically significant at a *p* value < 0.05.

## 3. Results

**Figure 1:**
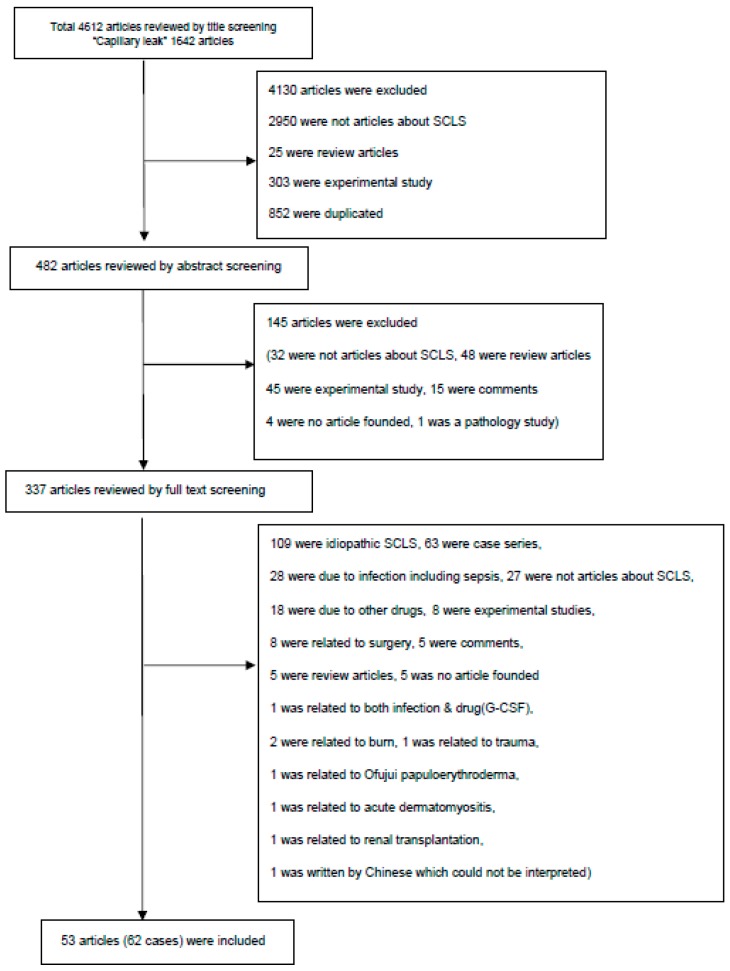
Flow chart of literature search.

The detailed process of literature search is presented in Figure 1. We identified 62 case reports on SCLS associated with cancer or anti-cancer agents. Patient data are summarized in Supplementary Table S2. Baseline characteristics are presented in Table 1. Forty-six % of the patients were aged over 50 years, while 21% were pediatric cases. There was a male preponderance, since 64.5% of cases were male. Sixty-one % of the patients had a hematologic malignancy, among which non-Hodgkin lymphoma was the most frequent (Table 1). SCLS was associated with cancer itself in 43.6%, associated with anti-cancer agents in 51.6% and occurred after bone marrow transplantation (BMT) in 4.8% of the patients. Among anti-cancer agents, granulocyte-colony stimulating factor (G-CSF) was the most frequent potentially causative drug (14.6%), followed by interleukin (IL)-2 (11.4%) (Table 2).

Main presenting symptoms and findings using physical and radiological examination of patients with SCLS included peripheral edema (67.7%), hypotension (32.2%), pleural effusion (29.0%), dyspnea (27.4%), ascites (22.7%), oliguria (22.7%), weight gain (21.0%), fever (17.7%) and pulmonary edema (11.3%) (Table 3). Laboratory findings showed that leukocytosis was observed in 40.1%, anemia in 48.0%, hemoconcentration in 63.6%, thrombocytopenia in 73.7% and hypoalbuminemia in 96.9% of the patients (Table 4).

Patients with SCLS were treated with steroids (59.7%), volume replacement (33.8%), diuretics (24.2%), inotropes (9.6%), methylxanthines (12.8%), β2 agonists (4.8%), intravenous immunoglobulins (IVIG) (3.2%), chemotherapeutic or immunosuppressive agents (11.3%) and procedures including dialysis or fluid drainage (8.0%) (Table 5). Twenty-nine % of patients with SCLS received a single therapy including steroids (19.4%), volume replacement (6.4%) and methylxanthines (3.2%) while 33.9% received two kinds of therapy, and 22.6% received more than three kinds of therapy with various combinations of the above mentioned therapies (Supplementary Table S3). 

**Figure 2:**
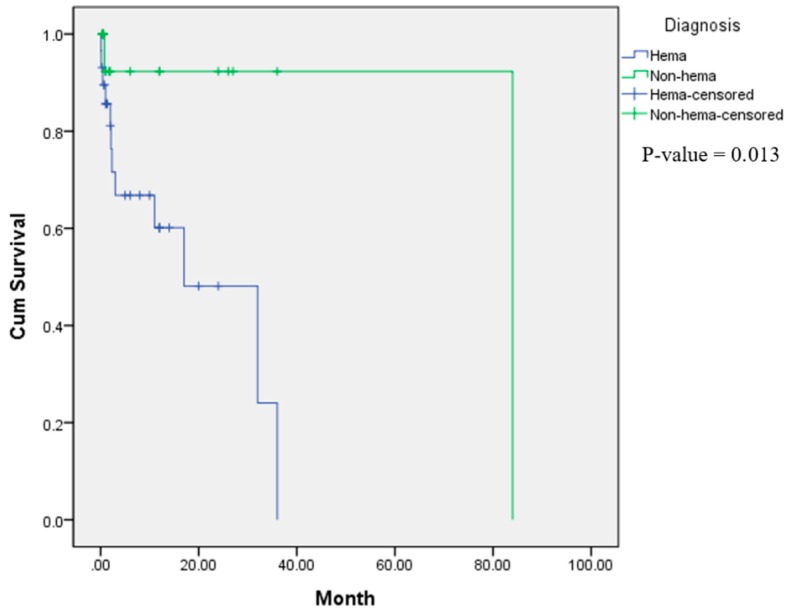
Kaplan-Meier analysis showing there was a decreased survival rate in systemic capillary leak syndrome patients with hematologic malignancies (*p* = 0.013).

To identify factors associated with mortality of patients with SCLS, various factors (demographic, clinical and laboratory features, and treatment) were compared between the 43 patients who recovered and the 16 patients who died. Univariate analyses showed that there were no differences in age, sex, cause of SCLS, diagnosis of cancer (hematologic or non-hematologic malignancy), systolic or diastolic blood pressures, serum albumin levels, WBC or platelet counts, hemoglobin and hematocrit levels and the use of steroid therapy (Supplementary Table S4). However, hematologic malignancies were associated with an increased risk for mortality (hazard ratio (HR) 8.820, 95% confidence interval (CI) 1.126–69.063, *p* = 0.038) on univariate analysis (Table 6). Kaplan-Meier analysis demonstrated that there were no differences in the survival rate between SCLS patients according to sex, decreased serum albumin levels (<2.5 vs. >2.5 g/dL and <3.0 vs. >3.0 g/dL), hypotension, leukocytosis and the use of steroid therapy (*p* > 0.05), but hematologic malignancies were associated with a decreased survival in cancer patients with SCLS (*p* = 0.013) (Figure 2 and Supplementary Figure S1).

The cause of death was documented in 13 of the 16 patients and in four was directly associated with SCLS. Other causes included cancer relapse or disease progression (2 patients), cardiac amyloidosis (1 patients), cardiac and hepatic amyloidosis (1 patient), sepsis (3 patients), and pneumonia (1 patient) (Supplementary Table S5).

## 4. Discussion

Since Clarkson et al. first reported the phenomenon of SCLS [1], more than 250 cases have been reported [3]. However, the exact incidence of SCLS is unclear and has not been described in idiopathic or secondary forms. We believe that SCLS might have been underrecognized and underestimated in the past because of its non-specific symptoms and signs which can be misdiagnosed as septic shock, anaphylaxis or various kinds of angioedema such as episodic angioedema with eosinophilia syndrome (Gleich’s syndrome) and non-episodic angioedema with eosinophilia and nodules, eosinophilia, rheumatism, dermatitis and swelling (NERDS) [7,8]. Considering the paucity of SCLS, the physician’s awareness is essential to reduce the substantial morbidity and mortality associated with it and to provide timely therapy [8]. 

In addition, it is important to improve knowledge about the pathophysiology, presenting symptoms and signs, as well as appropriate diagnostic and therapeutic strategies. There have been some reports on the beneficial effect of IVIG to treat idiopathic SCLS [5,6]. In contrast, limited evidence is present regarding treatment possibilities in secondary SCLS.

Although no study has investigated soluble factors or diagnostic markers of cancer-related SCLS, some pathogenic molecules have been investigated in idiopathic SCLS and these include; (1) increased numbers of circulating CD25^+^ T cells and perivascular infiltrations of mononuclear cells with an increased number of CD8^+^ T lymphocytes, suggesting T cell activation [9,10,11]; (2) endothelial injury and apoptosis [12,13], (3) increased serum cytokine levels such as elevated serum chemokine (C-X-C motif) ligand (CXCL)10, chemokine ligand 2 (CCL2), IL-1β, IL-6, IL-8, IL-12 and tumor necrosis factor-α (TNF-α) [14,15] and (4) high levels of plasma vascular endothelial growth factor (VEGF) and angiopoietin-2 [16]. However, few mechanisms have been suggested in the pathogenesis of SCLS related to cancer or cancer-related drugs [17].

There have been scarce reports on SCLS associated with cancer or cancer treatment-related drugs and no guidelines have been established on its management. Therefore, our study is the first to perform a systematic analysis of all published case reports. Through documented case reports, we found that SCLS can develop as a first manifestation of cancer or during cancer, after the use of several cancer treatment-related drugs and after BMT. Therefore, clinicians should at least consider a differential diagnosis of SCLS in cancer patients during initial presentation and disease course.

The clinical presentation of SCLS in cancer patients appears to be similar to that observed in idiopathic forms, including edema, dyspnea, and ascites due to capillary leak, accompanied by hypoalbuminemia, and hypotension due to hypovolemia. Signs such as fever (17.7%) and leukocytosis (40.1%) suggest that inflammation might be one of the triggering factors. In most patients, there was hypoalbuminemia, but one patient (3%) had a normal albumin level and thus developed capillary leakage by another unknown mechanism. As expected, 63.6% of patients showed hemoconcentration.

Due to the rarity of SCLS, treatment strategies are mostly based on observational studies rather than randomized controlled clinical trials. Furthermore, as the exact pathophysiology of idiopathic or secondary SCLS is still largely unknown, the treatment of SCLS is mostly determined by empirical use of several drugs. In idiopathic SCLS, volume replacement using intravenous albumin, crystalloids, or colloids is recommended to treat acute episodes. It was suggested that the use of steroids cannot prevent the acute episodes in most patients and may actually be deleterious to patients experiencing more frequent attacks despite occasional success in some patients [2]. In our analysis, however, steroids were used in 60% of the patients, which did not affect mortality and patient survival. β2 agonists and theophylline have been used to prevent recurrences of idiopathic SCLS [2], but only a small percent of patients received these agents in our systematic review. In addition, IVIG has been used successfully in the treatment of idiopathic SCLS, but only two patients (3.2%) received IVIG in cancer-associated SCLS. These results indicate that treatment patterns of SCLS in cancer patients might differ from those used in idiopathic SCLS. There is a clear need to design clinical trials to determine the therapeutic efficacy of steroids, IVIG, β2 agonists and other agents in the management of cancer-associated SCLS, which will facilitate the establishment of treatment guidelines.

With respect to the prognosis of SCLS in cancer patients, we found that hematologic malignancies were associated with an increased risk for mortality. There were no other demographic, clinical or laboratory indicators to predict mortality in our analysis and the use of steroids did not affect the outcome. Although death as outcome was observed in 16 of the 59 patients (27.1%) in our analysis, death could be directly attributed to SCLS only in four of the 13 (30.8%) patients in which the cause of death was documented.

## 5. Conclusions

We analyzed the clinical and laboratory data, treatment patterns and patient survival in all published cases with cancer-associated SCLS. However, our systematic review may have the limitation of some missed case reports. Nevertheless, our data will give a relevant insight by promoting knowledge about SCLS in cancer patients. The therapeutic role of IVIG which has been shown to be effective in idiopathic CLS should also be evaluated in cancer-associated SCLS [18,19,20].

## Figures and Tables

**Table 1:** Age, sex, and diagnosis of cancer patients with systemic capillary leak syndrome.

Variables	Observed Number of Patients (%) among Total Number of Patients (*n* = 62)
Age (years)	
<10	5 (8.1%)
10–19	8 (12.9%)
20–29	1 (1.6%)
30–39	8 (12.9%)
40–49	11 (17.7%)
50–59	12 (19.4%)
>60	17 (27.4%)
Sex	
Male	40 (64.5%)
Female	22 (35.5%)
Diagnosis of cancer	
Hematologic malignancy	38 (61.3%)
Hodgkin lymphoma	4 (6.5%)
Non-Hodgkin lymphoma	14 (22.7%)
Multiple myeloma	8 (12.9%)
Hemophagocytic lymphohistiocytosis	3 (4.8%)
Acute lymphoblastic leukemia	2 (3.2%)
Acute myeloid leukemia	1 (1.6%)
Chronic myelocytic leukemia	2 (3.2%)
Plasma cell leukemia	1 (1.6%)
Malignant lymphoma of tonsil	1 (1.6%)
Malignant lymphoma of cervical cord	1 (1.6%)
Fanconi anemia	1 (1.6%)
Non-hematologic malignancy	24 (38.7%)
Renal cell carcinoma	4 (6.5%)
Colorectal cancer	4 (6.5%)
Pancreatic cancer	3 (4.8%)
Hepatic carcinoma	2 (3.2%)
Non-small cell lung cancer	2 (3.2%)
Breast cancer	2 (3.2%)
Pituitary adenoma	1 (1.6%)
Primitive neuroectodermal tumor	1 (1.6%)
Distal common bile duct cancer	1 (1.6%)
Nasopharyngeal cancer	1 (1.6%)
Ovarian cystic teratoma	1 (1.6%)
Sarcoma	1 (1.6%)
Myxofibroma of maxilla	1 (1.6%)

**Table 2:** Etiologies of cancer patients with systemic capillary leak syndrome.

Variables	Total Number of Patients (*n* = 62) Observed Number of Patients (%)
Cancer ^*^	27 (43.6%)
BMT-related GVHD	3 (4.8%)
Anti-cancer agents	32 (51.6%)
G-CSF	9 (14.6%)
Interleukin-2	7 (11.4%)
Denileukin diftitox	2 (3.2%)
Gemcitabine	2 (3.2%)
MINE regimen	2 (3.2%)
Gemcitabine + paclitaxel	1 (1.6%)
Doxorubicin	1 (1.6%)
Bortezomib	1 (1.6%)
Clofarabine	1 (1.6%)
Cyclosporin A	1 (1.6%)
Trastuzumab	1 (1.6%)
Busulfan + etoposide + nimustine	1 (1.6%)
Pemetrexed	1 (1.6%)
Oxaliplatin	1 (1.6%)
Oxaliplatin + capecitabine	1 (1.6%)

BMT: Bone marrow transplantation, GVHD: Graft-versus-host disease, G-CSF: Granulocyte-colony stimulating factor, MINE regimen: mitoguazone, ifosfamide, vinorelbine, etoposide. * “Cancer” refers to systemic capillary leak syndrome cases only due to cancer itself, excluding other factors such as GVHD or induced by chemotherapeutic agents.

**Table 3:** Clinical presentation of cancer patients with systemic capillary leak syndrome.

Clinical Presentation	Observed Number of Patients (%) among Total Number of Patients (*n* = 62)
General condition	
Edema	42 (67.7%)
Weight gain	13 (21.0%)
Malaise	5 (8.1%)
General weakness	3 (4.8%)
Skin rash	4 (6.4%)
Hot flushing	1 (1.6%)
Sweating	1 (1.6%)
Gum hypertrophy	1 (1.6%)
Weight loss	1 (1.6%)
Disturbance of consciousness	1 (1.6%)
Inflammation-related	
Fever	11 (17.7%)
Lymph node enlargement	3 (4.8%)
Otalgia	1 (1.6%)
Pulmonary	
Dyspnea	17 (27.4%)
Pleural effusion	18 (29.0%)
Pulmonary edema	7 (11.3%)
Tachypnea	5 (8.1%)
Hypoxemia	4 (6.4%)
Pulmonary hypertension	1 (1.6%)
Cardiovascular	
Hypotension	20 (32.2%)
Hypertension	3 (4.8%)
Pericardial effusion	6 (9.7%)
Tachycardia	5 (8.1%)
Bradycardia	1 (1.6%)
Chest pain	1 (1.6%)
Syncope	1 (1.6%)
Decreased exercise tolerance	1 (1.6%)
Pericarditis	1 (1.6%)
Gastrointestinal	
Ascites	14 (22.7%)
Nausea	3 (4.8%)
Vomiting	4 (6.4%)
Abdominal distention	3 (4.8%)
Diarrhea	2 (3.2%)
Hepatosplenomegaly	1 (1.6%)
Renal	
Oliguria	14 (22.7%)
Proteinuria	1 (1.6%)
Neurologic	
Dizziness	1 (1.6%)
Back discomfort	1 (1.6%)
Tremor	1 (1.6%)
Paresthesia	1 (1.6%)

**Table 4:** Laboratory findings of cancer patients with systemic capillary leak syndrome.

Laboratory Findings	Total Number of Patients (*n* = 62) Observed/Measured Number of Patients (%)
WBC count	
Leukocytosis (>15,000/μL)	11/27 (40.1%)
Leukopenia (<4000/μL)	9/27 (33.3%)
Normal (4000–15,000/μL)	7/27 (25.9%)
No information	35/62 (56.4%)
Hemoglobin	
Anemia (<12.0 g/dL)	12/25 (48.0%)
Polycythemia (>15.0 g/dL)	6/25 (24.0%)
Normal (12.0–15.0 g/dL)	7/25 (28.0%)
No information	37/62(60.0%)
Hematocrit	
Hemoconcentration (Hct > 41%)	7/11 (63.6%)
Decreased hematocrit (Hct < 30%)	2/11 (18.2%)
Normal (Hct 30–41%)	2/11 (18.2%)
No information	51/62 (82.3%)
Platelet count	
Thrombocytopenia (<150,000/μL)	14/19 (73.7%)
Normal (150,000–450,000/μL)	5/19 (26.3%)
No information	43/62 (69.4%)
Albumin	
Very low (<2.5 g/dL)	16/32 (50.0%)
Low (2.5–3.5 g/dL)	15/32 (46.9%)
Normal (>3.5 g/dL)	1/32 (3.1%)
No information	30/62 (48.4%)

WBC: white blood cell, Hct: hematocrit.

**Table 5:** Treatment of cancer patients with systemic capillary leak syndrome.

Treatment	Observed Number of Patients (%) among Total Number of Patients (*n* = 62)
Steroids	37 (59.7%)
Methylprednisolone	14 (22.7%)
Prednisolone	12 (19.4%)
Cortisone	4 (6.4%)
Hydrocortisone	3 (4.8%)
Dexamethasone	2 (3.2%)
Methylprednisolone → prednisolone	1 (1.6%)
Other steroids	1 (1.6%)
Volume replacement	21 (33.8%)
Fluid resuscitation	4 (6.4%)
Crystalloid/colloid	2 (3.2%)
Transfusion *	1 (1.6%)
Albumin	11 (17.7%)
Hydroxyethyl starch	1 (1.6%)
Fluid resuscitation + albumin	1 (1.6%)
Fluid resuscitation + transfusion + albumin + hydroxyethyl starch	1 (1.6%)
Diuretics	15 (24.2%)
Furosemide	6 (9.6%)
Other diuretics	9 (14.6%)
Inotropes	6 (9.6%)
Dopamine	1 (1.6%)
Norepinephrine	1 (1.6%)
Other vasopressors	4 (6.4%)
Methylxanthines	8 (12.8%)
Theophylline	6 (9.6%)
Aminophylline	2 (3.2%)
β2 agonists	3 (4.8%)
Terbutaline	2 (3.2%)
Terbutaline + tulobuterol	1 (1.6%)
Intravenous immunoglobulins	2 (3.2%)
Chemotherapeutic or immunosuppressive agents †	7 (11.3%)
Cyclosporine A	2 (3.2%)
Cyclophosphamide	1 (1.6%)
Melphalan	1 (1.6%)
Chloraminophene	1 (1.6%)
Bevacizumab	1 (1.6%)
Cyclosporine A + cyclophosphamide + melphalan	1 (1.6%)
Other agents	6 (9.6%)
Antibiotics (cefepime + vancomycin)	1 (1.6%)
Antihistamine	1 (1.6%)
Leukotriene antagonist (Montelukast^®^)	1 (1.6%)
Serine protease inhibitor (Ulinastatin^®^)	1 (1.6%)
Radiosone	1 (1.6%)
Naftazone	1 (1.6%)
Procedure	5 (8.0%)
SLEDD	1 (1.6%)
CRRT	1 (1.6%)
Pericardial/thoracic/ascites drainage	2 (3.2%)
Plasma exchange	1 (1.6%)

SLEDD: Slow extended daily dialysis, CRRT: Continuous renal replacement therapy. * Transfusion contains only blood-driven materials (e.g., packed red blood cell (RBC), platelet, or fresh frozen plasma (FFP)) except albumin. † Only contained cases used for the treatment of systemic capillary leak syndrome, not for chemotherapy.

**Table 6:** Factors associated with mortality in cancer patients with systemic capillary leak syndrome.

Variables	Univariate Cox Proportional Hazard Model		
	Hazard Ratio	95% CI	*p* Value
Age	1.005	0.978 to 1.032	0.739
Sex	1.293	0.415 to 4.024	0.657
Hematologic	8.820	1.126 to 69.063	0.038
Cancer-induced	0.498	0.161 to 1.543	0.227
Drug-induced	2.455	0.794 to 7.591	0.119
SBP	1.012	0.945 to 1.085	0.728
DBP	1.371	0.452 to 4.16	0.578
Hypotension	1.235	0.36 to 4.244	0.737
Alb	1.379	0.417 to 4.558	0.598
Alb < 2.5 g/dL	3.878	0.402 to 37.423	0.241
Alb < 3.0 g/dL	1.687	0.17 to 16.773	0.655
WBC	1.000	1 to 1	0.352
WBC > 15,000/μL	0.022	0 to 2908.101	0.526
Hemoglobin	1.059	0.863 to 1.301	0.582
Platelet	1.000	1 to 1	0.600
Steroid	0.561	0.169 to 1.858	0.344

SBP: Systolic blood pressure, DBP: Diastolic blood pressure, Alb: Albumin, WBC: White blood cell count, CI: confidence interval.

## Supplementary Materials

The following are available online at http://www.mdpi.com/2077-0383/7/11/418/s1, Table S1: Checklist summarizing compliance with PRISMA guidelines, Table S2: Summary profiles of cancer patients with systemic capillary leak syndrome, Table S3: Combination of treatment for cancer patients with systemic capillary leak syndrome, Table S4: Comparison of variables in cancer patients with systemic capillary leak syndrome with or without death, Table S5: Cause of death of cancer patients with systemic capillary leak syndrome, Figure S1: Kaplan-Meier analysis for survival rate in SCLS patients according to various variables.
