# Supplementary material for: Systemic Capillary Leak Syndrome (Clarkson Syndrome) in Cancer Patients: A Systematic Review
Source: J Clin Med. 2018 Nov 6;7(11):418. doi: 10.3390/jcm7110418 (PMC6262589; doi:10.3390/jcm7110418)

Supplementary Table S1. Checklist summarizing compliance with PRISMA guidelines.

Section/topic	#	Checklist item	Reported on page #
**TITLE**			
Title	1	Identify the report as a systematic review, meta-analysis, or both.	1
**ABSTRACT**			
Structured summary	2	Provide a structured summary including, as applicable: background; objectives; data sources; study eligibility criteria, participants, and interventions; study appraisal and synthesis methods; results; limitations; conclusions and implications of key findings; systematic review registration number.	2
**INTRODUCTION**			
Rationale	3	Describe the rationale for the review in the context of what is already known.	4-5
Objectives	4	Provide an explicit statement of questions being addressed with reference to participants, interventions, comparisons, outcomes, and study design (PICOS).	4-5
**METHODS**			
Protocol and registration	5	Indicate if a review protocol exists, if and where it can be accessed (e.g., Web address), and, if available, provide registration information including registration number.	N/A
Eligibility criteria	6	Specify study characteristics (e.g., PICOS, length of follow-up) and report characteristics (e.g., years considered, language, publication status) used as criteria for eligibility, giving rationale.	5-6
Information sources	7	Describe all information sources (e.g., databases with dates of coverage, contact with study authors to identify additional studies) in the search and date last searched.	5-6
Search	8	Present full electronic search strategy for at least one database, including any limits used, such that it could be repeated.	5-6
Study selection	9	State the process for selecting studies (i.e., screening, eligibility, included in systematic review, and, if applicable, included in the meta-analysis).	5-6
Data collection process	10	Describe method of data extraction from reports (e.g., piloted forms, independently, in duplicate) and any processes for obtaining and confirming data from investigators.	5-6

Data items	11	List and define all variables for which data were sought (e.g., PICOS, funding sources) and any assumptions and simplifications made.	5-6
Risk of bias in individual studies	12	Describe methods used for assessing risk of bias of individual studies (including specification of whether this was done at the study or outcome level), and how this information is to be used in any data synthesis.	N/A
Summary measures	13	State the principal summary measures (e.g., risk ratio, difference in means).	5-6
Synthesis of results	14	Describe the methods of handling data and combining results of studies, if done, including measures of consistency (e.g., I^2) for each meta-analysis.	5-6

**Section/topic**	**#**	**Checklist item**	**Reported on page #**
Risk of bias across studies	15	Specify any assessment of risk of bias that may affect the cumulative evidence (e.g., publication bias, selective reporting within studies).	N/A
Additional analyses	16	Describe methods of additional analyses (e.g., sensitivity or subgroup analyses, meta-regression), if done, indicating which were pre-specified.	N/A
**RESULTS**			
Study selection	17	Give numbers of studies screened, assessed for eligibility, and included in the review, with reasons for exclusions at each stage, ideally with a flow diagram.	7-8
Study characteristics	18	For each study, present characteristics for which data were extracted (e.g., study size, PICOS, follow-up period) and provide the citations.	Supplementary tables
Risk of bias within studies	19	Present data on risk of bias of each study and, if available, any outcome level assessment (see item 12).	N/A
Results of individual studies	20	For all outcomes considered (benefits or harms), present, for each study: (a) simple summary data for each intervention group (b) effect estimates and confidence intervals, ideally with a forest plot.	Supplementary tables
Synthesis of results	21	Present results of each meta-analysis done, including confidence intervals and measures of consistency.	N/A
Risk of bias across studies	22	Present results of any assessment of risk of bias across studies (see Item 15).	N/A
Additional analysis	23	Give results of additional analyses, if done (e.g., sensitivity or subgroup analyses, meta-regression [see Item 16]).	N/A
**DISCUSSION**			

Summary of evidence	24	Summarize the main findings including the strength of evidence for each main outcome; consider their relevance to key groups (e.g., healthcare providers, users, and policy makers).	11
Limitations	25	Discuss limitations at study and outcome level (e.g., risk of bias), and at review-level (e.g., incomplete retrieval of identified research, reporting bias).	11
Conclusions	26	Provide a general interpretation of the results in the context of other evidence, and implications for future research.	11
**FUNDING**			
Funding	27	Describe sources of funding for the systematic review and other support (e.g., supply of data); role of funders for the systematic review.	12

From: Moher D, Liberati A, Tetzlaff J, Altman DG, The PRISMA Group (2009). Preferred Reporting Items for Systematic Reviews and Meta-Analyses: The PRISMA Statement. PLoS Med 6(6): e1000097. doi:10.1371/journal.pmed1000097

Supplementary Table S2. Summary profiles of cancer patients with systemic capillary leak syndrome

Ref. No.	Author, year	Age/ Sex	Presenting symptoms	Etiology of SCLS	Cancer diagnosis	Blood pressure (mmHg)	Albumin (g/dL)	Treatment	Outcome	Cause of Death
1	Pothen, et al., 2014	75/F	Hypotension, leg edema, anasarca, renal failure, hypoalbuminemia	Cancer	Diffuse large B-cell lymphoma	90/60	2.9	Theophylline treatment (200 mg/day)	Alive	-
2	Casadei Gardini, et al., 2013	65/F	Pulmonary hypertension	Drug (gemcitabine, nab-paclitaxel)	Ductal carcinoma (pT3 N0 M0, stage IIa), ovarian cancer	-	0.6	High-dose diuretics (furosemide 250 mg daily) combined with cortisone (40 mg methylprednisolone)	Alive (Relapse)	
3	Zhang, et al., 2013	59/F	Generalized edema, severe hypotension	Drug (trastuzumab)	Invasive carcinoma of the breast	-	3	Radiosone, diuretics, continuous thoracocentesis & pericardiocentesis	Alive	-
4	Lourdes et al., 2012	71/F	Lower extremity and facial swelling, nausea, vomiting, diarrhea	Cancer	ALK-negative anaplastic large cell lymphoma	70/-	3	Methylprednisolone 90 mg twice daily	Died	Cancer relapse
5	Ahn et al., 2012	50/F	Fever, progressive abdominal distention, nausea, bilious emesis, malaise, weakness	Cancer	Stage IV rectal cancer	69/17	< 1	Crystalloid, colloid, and vasopressors	Alive	-
6	Maroz et al., 2012	74/M	Pericardial and pleural effusion, severe edema of the face, abdominal wall, and lower and upper extremities	Drug (pemetrexed therapy)	Non–small cell lung cancer	-	2.0	Fluid resuscitation, discontinuation of pemetrexed	Alive	-
7	Kai-Feng et al., 2011	46/M	Hypotension, hypoproteinemia	Drug (Interleukin-11)	Hepatic carcinoma	102/61	1.8	Fluid resuscitation, transfusion, infusion of hydroxyethyl starch and albumin	Alive	-
7	Kai-Feng et al., 2011	66/M	Dyspnea with bilateral edema of the hands	Drug (Interleukin-11)	Hepatic carcinoma	93/59	-	Fluid resuscitation	Alive	-
8	Bencsath et	43/F	Hypotension, unexplained shock	Cancer	Lobular carcinoma of the breast	Hypotens	-	Terbutaline and theophylline, albumin	Alive	-

Ref. No.	Author, year	Age/ Sex	Presenting symptoms	Etiology of SCLS	Cancer diagnosis	Blood pressure (mmHg)	Albumin (g/dL)	Treatment	Outcome	Cause of Death
	al., 2011					ion		infusion, montelukast		
9	Umemoto et al., 2011	13/M	High fever, dyspnea, hypoxemia, pulmonary edema	Cancer	ALK+anaplastic large cell lymphoma	-	-	High dose methylprednisolone	Alive	-
10	Krzesiński et al., 2010	71/M	General weakening, decreased exercise tolerance, symmetric crural edema	Drug (doxorubicin)	Myeloma multiplex IgA (stage III)	100/80	2.9	Methylprednisolone therapy (Solu-medrol) at high doses (initially 1.5mg/kg /day)	Alive	-
11	Hsiao et al., 2010	65/F	Ascites, general anasarca	Drug (bortezomib)	Relapsed multiple myeloma	-	-	Dexamethasone	Died	Cardiac amyloidosis
12	Baytan et al., 2010	10/F	Hypotension, periorbital edema, oliguria, dyspnea	Drug (clofarabine)	Refractory acute lymphoblastic leukemia	70/50	-	Albumin, immunoglobulin	Died (Relapse)	SCLS
13	Ingegnoli et al., 2009	61/M	Rapidly progressing shortness of breath, profuse sweating, leg edema, severe fatigue, otalgia	Cancer	Nasopharyngeal carcinoma	-	-	High-dose prednisone (60 mg/day, subsequently gradually tapered), furosemide (25mg/day)	Alive	-
14	Zhang et al., 2009	49/M	Abdomen distention, hypotension	Cancer	Metastatic liver sarcoma	90/60	3	Hydroxyethyl starch	Alive	-
15	Avarbock et al., 2008	53/M	Dizziness and brief syncope episode, generalized muscle aches, respiratory distress	Drug (denileukin diftitox)	Cutaneous T-cell lymphoma	-	2.1	Albumin, vancomycin, cefepime, vasopressors	Died	SCLS
16	Baron et al., 2006	55/M	Generalized edema, dyspnea	Cancer	Adenocarcinoma of the pancreas	-	2.0	Loop diuretics and high-dose IV steroids	Alive	
17	Deeren et al.,	66/F	Weight gain, urine output decreased,	Drug (G-CSF)	Stage IIIB diffuse large B-cell	98/45	2.1	Corticosteroids, dialysis	Died	SCLS

Ref. No.	Author, year	Age/ Sex	Presenting symptoms	Etiology of SCLS	Cancer diagnosis	Blood pressure (mmHg)	Albumin (g/dL)	Treatment	Outcome	Cause of Death
	2005		lower limb edema		lymphoma				(Relapse)	
18	Biswas et al., 2004	58/M	Dyspnea, generalized edema	Cancer	Adenocarcinoma of the pancreatic uncinate	-	-	High-dose diuretic therapy, hydrocortisone 100 mg four times a day	Alive	-
19	Pulkkanen et al., 2003	50/M	Mild dyspnea during exercise, pleural effusion	Drug (gemcitabine)	Renal cell carcinoma	-	2.2	IV furosemide and oral prednisolone (starting with 30 mg per day and tapered over the next 4 weeks)	Alive	-
20	Rechner et al., 2003	57/M	Respiratory distress, hypotension, oliguria, edema, fever	Drug (G-CSF)	IgG kappa myeloma	+	-	Crystalloid, noradrenaline infusion, prednisolone 500 mg twice daily	Alive	-
20	Rechner et al., 2003	37/M	Anuria, edema, hypotension	Drug (G-CSF)	Chronic myeloid leukemia	-	-	Albumin. methyl prednisolone	Died	-
21	Vigneau et al.,2002	33/M	Shock with generalized edema, diffuse pain, transient anuria, proteinuria, weight loss, worsening cardiac function, pericardial effusion	Cancer	Multiple myeloma status post APBSCT			Fluid resuscitation, corticosteroids, theophylline, cyclophosphamide, cyclosporin A, plasma exchanges, naftazone, melphalan	Died	Cardiac and hepatic amyloidosis
22	De Pas et al., 2001	51/M	Worsening dyspnea, diffuse swelling, weight gain, hypotension	Drug (gemcitabine)	Stage III non-small-cell lung cancer	+	3.2	Furosemide, high dose dexamethasone	Alive	-
23	Meech et al., 2001	1/M	Fever, hepatosplenomegaly, peripheral edema, pleural effusion	Cancer	Anaplastic large cell lymphoma	-	-	High-dose IVIG, Methylprednisolone, cyclosporin A	Alive	-
24	Dagdemir et al., 2001	14/F	Fever, respiratory distress, hypotension	Drug (G-CSF)	Acute non-lymphoblastic leukemia (FAB-M2)	+	-	Methylprednisolone (1mg/kg/d)	Alive	-

Ref. No.	Author, year	Age/ Sex	Presenting symptoms	Etiology of SCLS	Cancer diagnosis	Blood pressure (mmHg)	Albumin (g/dL)	Treatment	Outcome	Cause of Death
25	Ghosh et al., 2001	42/M	Dyspnea, ascites, weight gain	Cancer	Plasma cell leukemia	-	3.3	Aminophylline, terbutaline, furosemide	Alive	-
26	Railan et al., 2000	80/M	Generalized erythroderma, chills, ascites, oliguria	Drug (denileukin diftitox)	Stage IB cutaneous T-cell lymphoma	-	2.6	Hydrocortisone	Alive	-
27	Jillella et al., 2000	43/F	Edema, fever, weight gain, anemia, thrombocytopenia, cervical LN enlargement	Cancer	Diffuse large B-cell non-Hodgkin lymphoma	-	2.4	-	Alive	-
27	Jillella et al., 2000	32/M	Generalized edema, fever, pleural effusion, ascites, multiple enlarged LNs	Cancer	Non-Hodgkin lymphoma	-	2.0	-	Died	Sepsis -
28	Al-Homaidhi et al., 1998	39/M	Weight gain, lower leg edema, fever, ascites, pleural effusion	Drug (G-CSF)	Stage II anodular sclerosing Hodgkin disease	-	-	Diuretics, albumin	Died	Pneumonia
28	Al-Homaidhi et al., 1998	44/M	Ascites, pleural effusion, leg swelling	Drug (G-CSF)	Stage IVB nodular sclerosing Hodgkin disease	-	-	Diuretics, albumin	Died	SCLS
29	Beermann et al., 1998	68/M	Shock with increase in hematocrit, leukocytosis, generalized edema	Cancer	Multiple myeloma	-	-	Mephalan, prednisolone	Died	Multiple myeloma
30	Takimoto et al., 1998	43/M	Systemic edema, pleural effusion, ascites	Cancer	Anaplastic large cell lymphoma	-	WNL	IV albumin, diuretics, antihistamines, prednisolone (30mg/day)	Alive (Relapse)	-
31	Hiraoka et al., 1995	67/F	Edema, oliguria, weight gain	Cancer	Multiple myeloma	122/78	3.1	Albumin, aminophylline	Alive	-
32	Abramov et al., 1995	33/F	Pitting edema, abdominal pain, vomiting, hypotension, tachycardia, oliguria	Cancer	Benign ovarian cystic teratoma	80/50	1.6	-	Died	-
33	Oeda et al., 1994	38/M	Dyspnea, pleural effusion	Drug (G-CSF)	Malignant lymphoma of tonsil	-	2.7	Methylprednisolone pulse	Alive	-

Ref. No.	Author, year	Age/ Sex	Presenting symptoms	Etiology of SCLS	Cancer diagnosis	Blood pressure (mmHg)	Albumin (g/dL)	Treatment	Outcome	Cause of Death
33	Oeda et al., 1994	38/M	Lower leg edema, hypotension, fever	Drug (G-CSF)	Malignant lymphoma of cervical cord	+	2.4	Methylprednisolone pulse	Alive	-
34	Dereure et al., 1994	66/F	Cutaneous edema, pericardial effusion, pleural effusion	Cancer	Sezary syndrome	-	-	-	Died	Sepsis
35	Kao et al., 1993	36/M	Nausea, vomiting, weakness, oliguria, low back discomfort	Cancer	Pituitary adenoma	-	-	Theophylline, prednisone	Died	Cancer
36	Ramon et al., 1986	49/M	Epigastric pain, hypertension, paresthesia, tremor, gum hypertrophy, facial edema, skin purpura	Drug (Cyclosporin A)	Sezary syndrome	-	-	Cyclosporine discontinuation	Alive	-
37	Jeong et al., 2014	41/F	Hypotension, bradycardia, hypoxia	Cancer	Distal common bile duct cancer	60/20	1.6	Theophylline, hydrocortisone	Alive	-
38	Andres et al., 2000	83/F	Edema	Cancer	Follicular non-Hodgkin lymphoma	-	-	Corticosteroid, chloraminophene	Alive	-
39	Benjelloun et al., 2014	63/M	Edema, pleural effusion, ascites, pericarditis	Cancer	Waldenstrom disease	-	1.2			-
40	Maubec et al., 2001	16/F	Painful and massive subcutaneous inflammatory edema	Drug (MINE regimen)	Hodgkin disease	-	-		Alive	
40	Maubec et al., 2001	17/M	Painful and massive subcutaneous inflammatory edema	Drug (MINE regimen)	Hodgkin disease	-	-		Alive	
41	Kobori et al., 2002	53/M	Edema, weight gain, pleural effusion, ascites	Drug (IL-2)	Metastatic renal cell carcinoma	-	2.8		Alive	-
41	Kobori et al., 2002	54/M	Edema, weight gain, pleural effusion, ascites	Drug (IL-2)	Metastatic renal cell carcinoma	-	2.7		Died	-
42	Ozawa et al.,	57/F	Edema, hypovolemic shock,	Cancer	Rectal cancer	-	1.5	Fluid resuscitation, CRRT, theophylline, tulobuterol,	Alive	-

Ref. No.	Author, year	Age/ Sex	Presenting symptoms	Etiology of SCLS	Cancer diagnosis	Blood pressure (mmHg)	Albumin (g/dL)	Treatment	Outcome	Cause of Death
	2015		hypoalbuminemia					prednisolone		
43	Funke et al., 1994	19/M	Edema, weight gain,	Cancer	Chronic myelogenous leukemia	-	3.8	Furosemide. prednisone, cyclophosphamide	Alive	-
44	Emminger et al., 1990	20/M	Weight gain, pleural effusion, peripheral edema	Drug (G-CSF)	Primitive neuroectodermal tumor	-	-	Prednisone, hydration	Died	Sepsis
45	Ohta et al., 1994	7M/M	Hypovolemic shock, pulmonary edema, pleural effusion, ascites	Drug (IL-2)	Hemophagocytic lymphohistiocytosis	-	-	High-dose methylprednisolone	Died	-
45	Ohta et al., 1994	2/M	Acute pulmonary edema	Drug (IL-2)	Hemophagocytic lymphohistiocytosis	-	-	High-dose methylprednisolone	Alive	-
46	Morino et al., 2003	4/F	Fever, ascites, facial swelling, tachycardia, tachypnea, enlarged cervical LNs, pleural effusion	Cancer	Hemophagocytic syndrome	118/46	2.7	Albumin, oral prednisolone, methylprednisolone pulse, cyclosporin A	Alive	-
47	Gyger et al., 2005	48/M	Dyspnea, lower leg edema, pleural effusions, pericardial effusions	GVHD	Multiple myeloma status post APBSCT	-	-	Prednisone	Alive	-
47	Gyger et al., 2005	46/F	Edema, dyspnea, chest pain, weight gain, pericardial effusion	GVHD	Multiple myeloma status post APBSCT	-	-	Prednisone	Alive	-
48	Takahashi et al., 1992	14/F	Weight gain, edema, pulmonary edema	Drug (busulfan, etoposide, nimustine)	Acute lymphoblastic leukemia	-	-	IV diuretics, albumin, methylprednisolone	Alive (Relapse)	-
49	Singh et al., 2013	13/F	Hypotension, abdominal pain, pleural effusion, ascites, pericardial effusion	Cancer	Myxofibroma of maxilla suspected Meigs' syndrome	66/45	2.5	IV vasopressors, ascites drainage, pericardial effusion drainage, pleural tapping	Alive	-
50	Sculier et. al., 1988	58/F	Weight gain, erythroderma, purpura, diarrhea, oliguria, hypotension,	Drug (IL-2)	Hypernephroma	75/-	-	Furosemide, low-dose dopamine (2mcg/kg/min),	Alive	-

Ref. No.	Author, year	Age/ Sex	Presenting symptoms	Etiology of SCLS	Cancer diagnosis	Blood pressure (mmHg)	Albumin (g/dL)	Treatment	Outcome	Cause of Death
			pulmonary edema					high-dose corticosteroid	(Relapse)	
51	Anderson et al., 2015	63/M	Dyspnea, tachycardia, tachypnea, hypotension, hypoxemia, pulmonary edema, anuria	Drug (oxaliplatin)	Colorectal cancer	-	2.4	Corticosteroids (stress dose), vasopressors	Alive	-
52	Liu et al., 2016	61/M	Tachypnea, muscular pain, fullness of the abdomen, edema, flushing	Drug (oxaliplatin + capecitabine)	Sigmoid cancer	111/56	2.97	Fluid resuscitation, furosemide, albumin, methylprednisolone, ascites drainage	Alive	
53	Yabe et al., 2010	6/M	Posterior reversible encephalopathy syndrome, mild systemic edema, grade 2 acute graft-versus-host disease of the skin and gastrointestinal tract, consciousness disturbance, tachypnea, tachycardia, massive pleural effusion, ascites, hypotension, negative central venous pressure, anuria	GVHD	Fanconi anemia status post APBSCT	+	-	Prednisolone (1mg/kg daily), ulinastatin (10 000 units/kg daily), albumin (0.8 g/kg every other day), IV bevacizumab (5mg/kg)	Alive	-

SCLS: systemic capillary leak syndrome, ALK: Anaplastic lymphoma kinase, CRRT: Continuous renal replacement therapy, G-CSF: Granolocyte colony stimulating factor, GVHD: Graft-versus-host disease, APBSCT: Autologous peripheral blood stem cell transplantation, MINE: mitoguazone, ifosfamide, vinorelbine,etoposide,
IV: intravenous, LN; lymph node

References

1. Pothen L, Rouvière H, Poncin R, Michaux L, Damoiseau P, Lambert M. Systemic capillary leak syndrome revealing a diffuse large B-cell lymphoma. Acta Clin Belg 2014;69:305–8.

2. Casadei Gardini A, Aquilina M, et al. Separate episodes of capillary leak syndrome and pulmonary hypertension after adjuvant gemcitabine and three years later after nab-paclitaxel for metastatic disease. BMC Cancer 2013;13:542.

3. Zhang F, Yang J, Li Z. Trastuzumab-Induced Systemic Capillary Leak Syndrome in a Breast Cancer Patient. Pathol Oncol Res 2014;20:435–7.

4. Lourdes LS, Al-Quran SZ, Dang NH, Markham MJ. Systemic Capillary Leak Syndrome as an Initial Presentation of ALK-Negative

Anaplastic Large Cell Lymphoma. Case Rep Hematol 2012;2012:954201.

5. Ahn J, Odom SR, Saillant N, Markham MJ. Capillary leak syndrome and abdominal compartment syndrome from occult rectal malignancy. Am Surg 2012;78:443–5.

6. Maroz N, Weiner ID. Secondary capillary leak syndrome related to pemetrexed exposure. Am J Kidney Dis 2012;59:582–3.

7. Kai-Feng W, Hong-Ming P, Hai-Zhou L, Li-Rong S, Xi-Yan Z. Interleukin-11-induced capillary leak syndrome in primary hepatic carcinoma patients with thrombocytopenia. BMC Cancer 2011;11:204.

8. Bencsath KP, Reu F, Dietz J, Hsi ED, Heresi GA. Idiopathic systemic capillary leak syndrome preceding diagnosis of infiltrating lobular carcinoma of the breast with quiescence during neoadjuvant chemotherapy. Mayo Clin Proc 2011;86:260–1.

9. Umemoto T, Watanabe T, Ogose T, et al. Capillary leak syndrome: initial presentation in a patient with ALK+anaplastic large cell lymphoma associated with increased levels of serum cytokines. Leuk Lymphoma 2011;52:1139–42.

10. Krzesiński P, Wierzbowski R, Gielerak G, et al. Impedance cardiography in the diagnosis of capillary leak syndrome caused by doxorubicin therapy in a patient with myeloma multiplex. Cardiol J 2010;17:88–91.

11. Hsiao SC, Wang MC, Chang H, Pei SN. Recurrent capillary leak syndrome following bortezomib therapy in a patient with relapsed myeloma. Ann Pharmacother 2010;44:587–9.

12. Baytan B, Ozdemir O, Gunes AM, Dönmez O. Clofarabine-induced capillary leak syndrome in a child with refractory acute lymphoblastic leukemia. J Pediatr Hematol Oncol 2010;32:144–6.

13. Ingegnoli F, Sciascera A, D'Ingianna E, Fantini F. Systemic sclerosis, capillary leak syndrome and nasopharyngeal carcinoma: an unusual association or paraneoplastic manifestations? Rheumatology (Oxford) 2009;48:201–2.

14. Zhang W, Wang K, Qian X, et al. Abdominal compartment syndrome associated with capillary leak syndrome after liver transplantation. Transplant Proc 2009;41:3927–30.

15. Avarbock AB, Loren AW, Park JY, et al. Lethal vascular leak syndrome after denileukin diftitox administration to a patient with cutaneous gamma/delta T-cell lymphoma and occult cirrhosis. Am J Hematol 2008;83:593–5.

16. Baron D, Mayo A, Kluger Y. Gemcitabine-induced chronic systemic capillary leak syndrome: a life-threatening disease. Clin Oncol (R Coll Radiol) 2006;18:90–1.

17. Deeren DH, Zachee P, Malbrain ML. Granulocyte colony-stimulating factor-induced capillary leak syndrome confirmed by extravascular lung water measurements. Ann Hematol 2005;84:89–94.

18. Biswas S, Nik S, Corrie PG. Severe gemcitabine-induced capillary-leak syndrome mimicking cardiac failure in a patient with advanced pancreatic cancer and high-risk cardiovascular disease. Clin Oncol (R Coll Radiol) 2004;16:577–9.

19. Pulkkanen K, Kataja V, Johansson R. Systemic capillary leak syndrome resulting from gemcitabine treatment in renal cell carcinoma: a case report. J Chemother 2003;15:287–9.

20. Rechner I, Brito-Babapulle F, Fielden J. Systemic capillary leak syndrome after granulocyte colony-stimulating factor (G-CSF). Hematol J 2003;4:54–6.

21. Vigneau C, Haymann JP, Khoury N, Sraer JD, Rondeau E. An unusual evolution of the systemic capillary leak syndrome. Nephrol Dial Transplant 2002;17:492–4.

22. De Pas T, Curigliano G, Franceschelli L, Catania C, Spaggiari L, de Braud F. Gemcitabine-induced systemic capillary leak syndrome. Ann Oncol 2001;12:1651–2.

23. Meech SJ, McGavran L, Odom LF, et al. Unusual childhood extramedullary hematologic malignancy with natural killer cell properties that contains tropomyosin 4--anaplastic lymphoma kinase gene fusion. Blood 2001;98:1209–16.

24. Dagdemir A, Albayrak D, Dilber C, Totan M. G-CSF related capillary leak syndrome in a child with leukemia. Leuk Lymphoma 2001;42:1445–7.

25. Ghosh K, Madkaikar M, Iyer Y, Pathare A, Jijina F, Mohanty D. Systemic capillary leak syndrome preceding plasma cell leukaemia. Acta Haematol 2001;106:118–21.

26. Railan D, Fivenson DP, Wittenberg G. Capillary leak syndrome in a patient treated with interleukin 2 fusion toxin for cutaneous T-cell lymphoma. J Am Acad Dermatol 2000;43:323–4.

27. Jillella AP, Day DS, Severson K, Kallab AM, Burgess R. Non-Hodgkin's lymphoma presenting as anasarca: probably mediated by tumor necrosis factor alpha (TNF-alpha). Leuk Lymphoma 2000;38:419–22.

28. Al-Homaidhi A, Prince HM, Al-Zahrani H, Doucette D, Keating A. Granulocyte-macrophage colony-stimulating factor-associated histiocytosis and capillary-leak syndrome following autologous bone marrow transplantation: two case reports and a review of the literature. Bone Marrow Transplant 1998;21:209–14.

29. Beermann W, Hörstrup KA, Will R. Systemic capillary leak syndrome. Am J Med 1998;105:554.

30. Takimoto Y, Imanaka F, Sasaki N, Nanba K, Kimura N. Gamma/delta T cell lymphoma presenting in the subcutaneous tissue and small intestine in a patient with capillary leak syndrome. Int J Hematol 1998;68:183–91.

31. Hiraoka E, Matsushima Y, Inomoto-Naribayashi Y, Nakata H, Nakamura A, Kawanami C. Systemic capillary leak syndrome associated with multiple myeloma of IgG kappa type. Intern Med 1995;34:1220–4.

32. Abramov Y, Galun E, Granat M, et al. Postpartum systemic capillary leak syndrome: a possible etiology. Acta Obstet Gynecol Scand 1995;74:395–8.

33. Oeda E, Shinohara K, Kamei S, Nomiyama J, Inoue H. Capillary leak syndrome likely the result of granulocyte colony-stimulating factor after high-dose chemotherapy. Intern Med 1994;33:115–9.

34. Dereure O, Portales P, Clot J, Guilhou JJ. Biclonal Sézary syndrome with capillary leak syndrome. Dermatology 1994;188:152–6.

35. Kao NL, Richmond GW, Luskin AT. Systemic capillary leak syndrome. Chest 1993;104:1637–8.

36. Ramon D, Betlloch I, Jimenez A, Botella R, Castells A, Alberola V. Remission of Sézary's syndrome with cyclosporin A. Mild capillary leak syndrome as an unusual side effect. Acta Derm Venereol 1986;66:80–2.

37. Jeong EK, Kim YK, Kim SH, Lee CH, Kim JS. Systemic capillary leak syndrome under general anesthesia: a case report. Korean J Anesthesiol 2014;66:462–6.

38. Andres E, Vinzio S, Ruellan A, Herbrecht R, Goichot B, Schlienger JL. Capillary hyperpermeability syndrome of the face. Presse Med 2000;29:1279–81.

39. Benjelloun S, Tlamçani I, Yahyaoui G, et al. Idiopathic capillary hyperpermeability syndrome revealing of Waldenström disease. Ann Biol Clin (Paris) 2014;72:236–40.

40. Maubec E, Oberlin O, Belhadj K, Roujeau JC. Subcutaneous inflammatory edema induced by MINE chemotherapy. Ann Dermatol Venereol 2001;128:534–7.

41. Kobori G, Maegawa M, Ushida H, et al. Two cases of renal cell carcinoma with ascites after interleukin-2 therapy. Hinyokika Kiyo 2002;48:455–7.

42. Ozawa T, Yamaguchi H, Kiyomatsu T, et al. A case report of idiopathic systemic capillary leak syndrome that occurred during the postoperative period of abdominoperineal resection for colorectal cancer. Int Surg 2015;100:58–62.

43. Funke I, Prümmer O, Schrezenmeier H, et al. Capillary leak syndrome associated with elevated IL-2 serum levels after allogeneic bone marrow transplantation. Ann Hematol 1994;68:49–52.

44. Emminger W, Emminger-Schmidmeier W, Peters C, et al. Capillary leak syndrome during low dose granulocyte-macrophage colony-stimulating factor (rh GM-CSF) treatment of a patient in a continuous febrile state. Blut 1990;61:219–21.

45. Ohta H, Yumara-Yagi K, Sakata N, Inoue M, Kawa-Ha K. Capillary leak syndrome in patients with hemophagocytic lymphohistiocytosis. Acta Paediatr 1994;83:1113–4.

46. Morino M, Kumakura R, Miura N, Sasaki N. Decreased hepatic uptake of gallium-67 citrate in haemophagocytic syndrome occurring concomitantly with capillary leak syndrome. Pediatr Radiol 2003;33:804–6.

47. Gyger M, Rosenberg A, Shamy A, et al. Vascular leak syndrome and serositis as an unusual manifestation of chronic graft-versus-host disease in nonmyeloablative transplants. Bone Marrow Transplant 2005;35:201–3.

48. Takahashi H, Sekiguchi H, Kai S, Ikuta K, Sasaki H, Matsuyama S. Recurrent pulmonary edema in a patient with acute lymphoblastic leukemia after syngeneic bone marrow transplantation. Rinsho Ketsueki. 1992;33:354-9.

49. Singh M, Singh S. An unusual case of sudden collapse in the immediate postoperative period in a young healthy female with myxofibroma of the maxilla. Case Rep Anesthesiol 2013;596758.

50. Sculier JP, Bron D, Verboven N, Klastersky J. Multiple organ failure during interleukin-2 administration and LAK cells infusion. Intensive Care Med 1988;14:666–7.

51. Anderson BJ, Peterson LL. Systemic capillary leak syndrome in a patient receiving adjuvant oxaliplatin for locally advanced colon cancer. J Oncol Pharm Pract 2016; 2016;22:725–8.

52. Liu Z, Li de C, Zhu YP. Interleukin-11-induced capillary leak syndrome companied with abdominal chylous leakage in primary sigmoid carcinoma patients with thrombocytopenia. J Cancer Res Ther 2015;11:1024.

53. Yabe H, Yabe M, Koike T, Shimizu T, Morimoto T, Kato S. Rapid improvement of life-threatening capillary leak syndrome after stem cell transplantation by bevacizumab. Blood 2010;115:2723–4.

Supplementary Table S3. Combination of treatment for cancer patients with systemic capillary leak syndrome

Treatment	Total number of patients (n=62)
	Number of patients (%)
**Single kind of therapy**	**18(29.0%)**
Steroids only	12(19.4%)
Volume replacement only	4(6.4%)
Methylxanthine only	2 (3.2%)
**Two kinds of therapy**	**21(33.9%)**
Steroids + Volume replacement	2(3.2%)
Steroids + Diuretics	6(9.7%)
Steroids + Inotropes	1 (1.6%)
Steroids + Methylxanthine	2(3.2%)
Steroids + Procedure*	1 (1.6%)
Steroids + Chemotherapeutic agents‡	2(3.2%)
Volume replacement + Diuretics	2(3.2%)
Volume replacement + Inotropes	1 (1.6%)
Volume replacement + Methylxanthine	1 (1.6%)
Volume replacement + Immunoglobulin	1 (1.6%)
Diuretics + Other agents†	1 (1.6%)
Inotropes + Procedure	1 (1.6%)
**Three kinds of therapy**	**8(12.9%)**
Steroids + Volume replacement + Diuretics	1 (1.6%)
Steroids + Volume replacement + Inotropes	1 (1.6%)
Steroids + Volume replacement + Chemotherapeutic agents	1 (1.6%)
Steroids + Diuretics + Inotropes	1 (1.6%)
Steroids + Diuretics + Chemotherapeutic agents	1 (1.6%)
Steroids + Immunoglobulin + Chemotherapeutic agents	1 (1.6%)
Volume replacement + Inotropes + Other agents	1 (1.6%)
Diuretics + Methylxanthine + β2 agonists	1 (1.6%)
**Four kinds of therapy**	**4(6.5%)**
Steroids + Volume replacement + Diuretics + Other agents	1 (1.6%)
Steroids + Volume replacement + Chemotherapeutic agents + Other agents	1 (1.6%)
Steroids + Volume replacement + Diuretics + Procedure	1 (1.6%)
Volume replacement + Methylxanthine + β2 agonist + Other agents	1 (1.6%)
**More than five kinds of therapy**	**2(3.2%)**
Steroids + Volume replacement+ Methylxanthine + β2 agonist+ Procedure	1 (1.6%)
Steroids + Volume replacement + Methylxanthine + Chemotherapeutic agents + Other agents + Procedure	1 (1.6%)
**No information about treatment**	**9(14.5%)**

*Procedure contains dialysis, continuous renal replacement therapy, pericardial/thoracic/ascites drainage or plasma exchange.

†Other agents contain all agents except steroid, diuretics, inotropes, methylxanthine, β2 agonist, immunoglobulin, and chemotherapeutic agents.

‡ Only contained cases used for the treatment of systemic capillary leak syndrome, not for chemotherapy

Supplementary Table S4. Comparison of variables in cancer patients with systemic capillary leak syndrome with or without death

**Valuables**	**Alive (n=43)***	**Death (n=16)***	***P* value**
Age (years) ± range	44.5±22.8	43.3±21.6	0.853
Sex (M/F)	27/16	11/5	0.766
Cause (cancer-itself‡/drugs §)	19/21	6/9	0.764
Diagnosis (hematologic/non-hematologic)	28/15	10/6	0.073
Follow up duration† (months) ± range	8.1±9.7	13.7±23.5	0.418
Hypotension vs normal or hypertension)	15/26	4/12	0.537
SBP (mmHg) ± range	91.4±21.4	79.3±16.2	0.389
DBP (mmHg) ± range	52.2±21.1	47.5±3.5	0.768
Albumin (g/dL) ± range	2.5±0.7	2.4±0.4	0.830
WBC (/μl) ± range	14214±13650	4150.0±5727	0.320
Hemoglobin (g/dl) ± range	12.2±5.5	13.5±4.8	0.611
Hematocrit (%) ± range	44.2±11.9	67.0±0.0	0.106
Platelet (/μl) ± range	75563±81010	46500.0±54447	0.633
Steroid (use/not use)	27/13	10/5	1.000

SBP : Systolic blood pressure, DBP: Diastolic blood pressure, WBC: White blood cell count

*Patient survival was reported in 60 of total 62 case report patients.

†Duration was the follow up months after initial diagnosis of systemic capillary leak syndrome in the articles.

‡Cause of systemic capillary leak syndrome induced only by cancer- itself, not induced by drugs, infection or bone marrow transplantation.

§ Drugs which caused systemic capillary leak syndrome means anti-cancer agents used to treat cancer. See Supplementary Table E4.

Supplementary Table S5. Cause of death of cancer patients with systemic capillary leak syndrome.

**Cause of death**	**No**
Cancer relapse	2
Cardiac amyloidosis	1
Cardiac + hepatic amyloidosis	1
Systemic capillary leak syndrome	4
Sepsis	3
Pneumonia	1
Multiple myeloma	1
**Total number**	13/62

Supplementary Figure S1 (a) Kaplan-Meier analysis showing there is no difference in survival rate between systemic capillary leak syndrome patients with death and those without according to sex (1: male, 2: female) (p=0.6409)

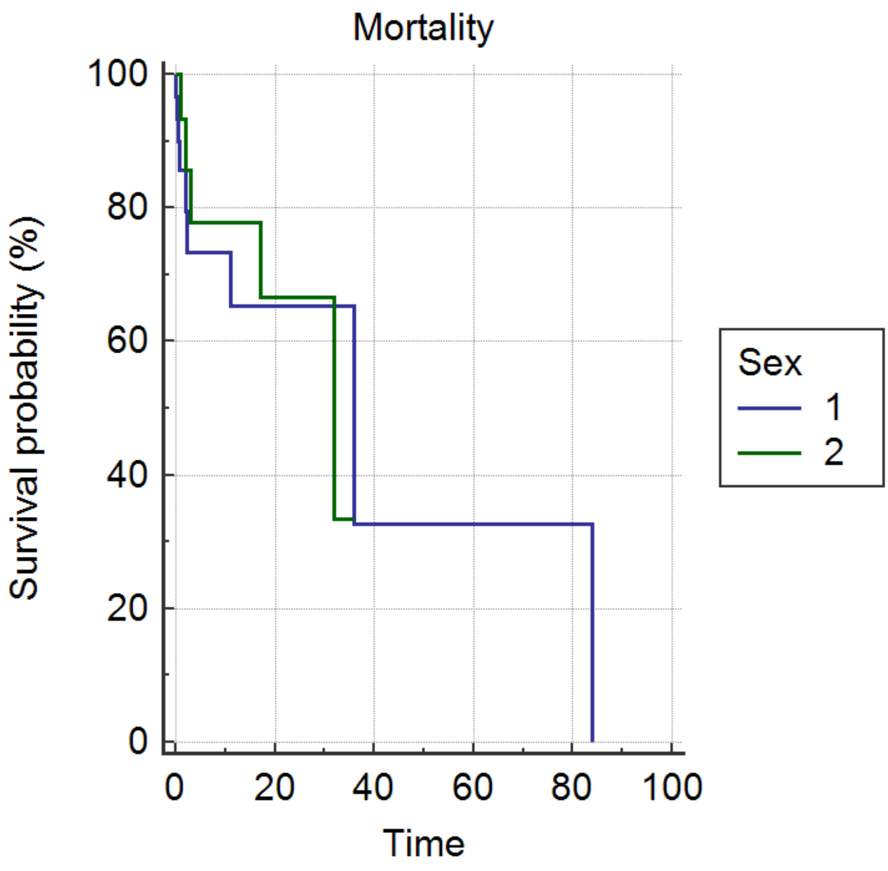

Supplementary Figure S1(b) Kaplan-Meier analysis showing there is no difference in survival rate between systemic capillary leak syndrome patients with death and those without according to serum albumin levels (1: < 2.5 vs.2: >2.5 g/dL) (p=0.193).

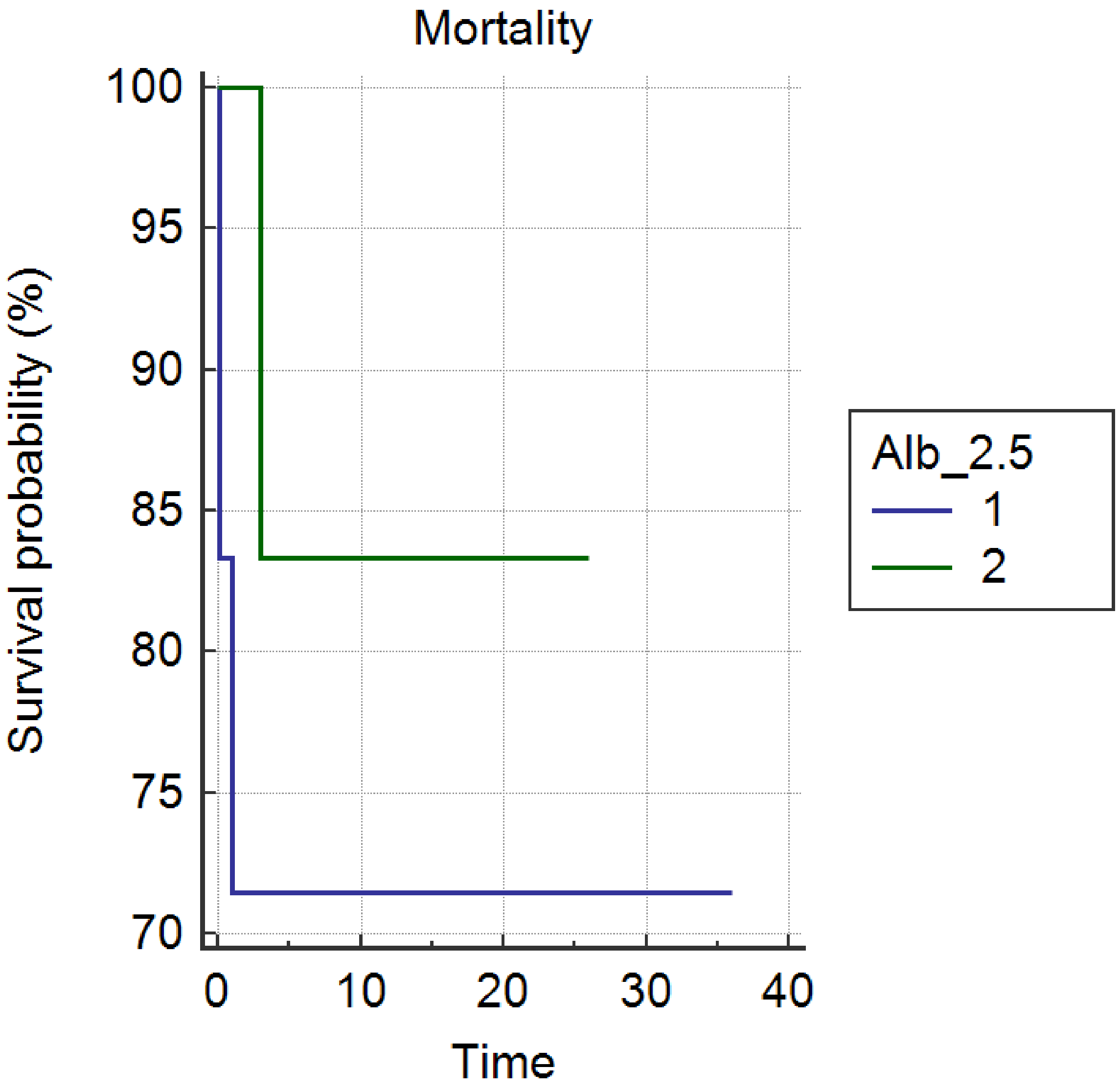

Supplementary Figure S1(c) Kaplan-Meier analysis showing there is no difference in survival rate between systemic capillary leak syndrome patients with death and those without according to serum albumin levels (1: < 3.0 vs.2: >3.0 g/dL) (p=0.614).

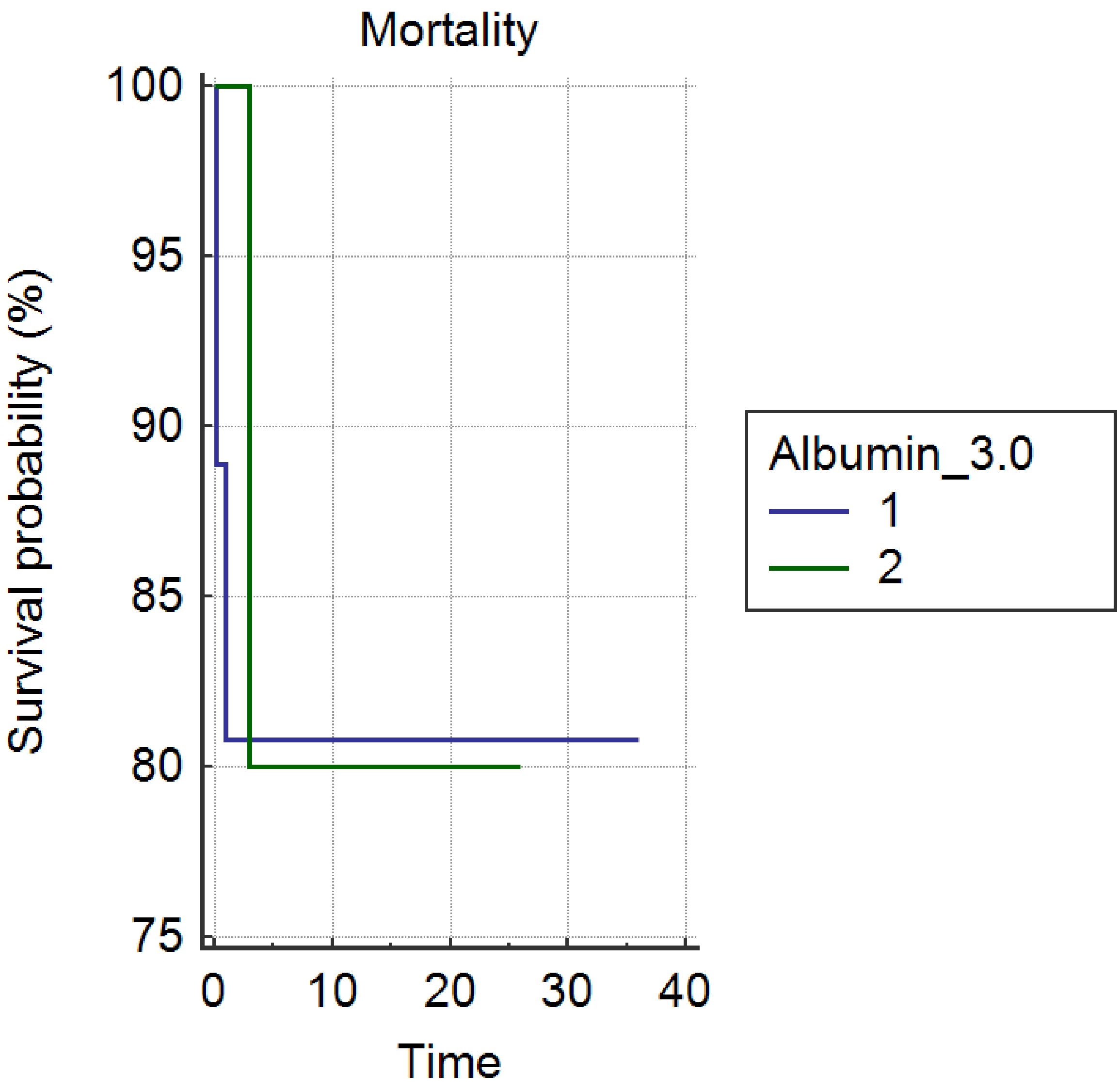

Supplementary Figure S1(d) Kaplan-Meier analysis showing there is no difference in survival rate between systemic capillary leak syndrome patients with death and those without according to cause of SCLS (1: cancer itself and 2: drug-associated) (p=0.117).

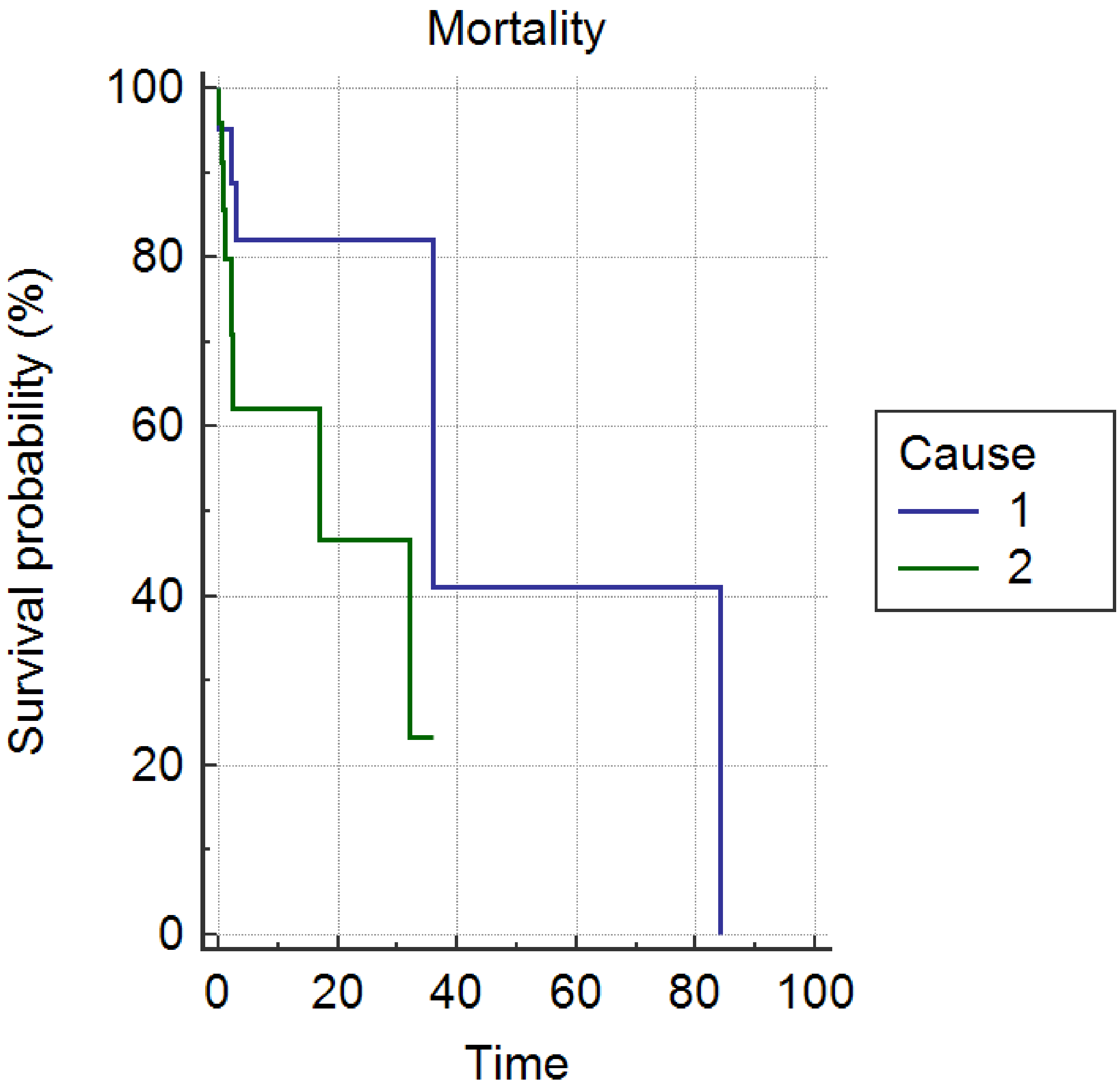

Supplementary Figure S1(e) Kaplan-Meier analysis showing there is no difference in survival rate between systemic capillary leak syndrome patients with death and those without according to the presence of hypotension (1: hypotension and 2: no hypotension) (p=0.758).

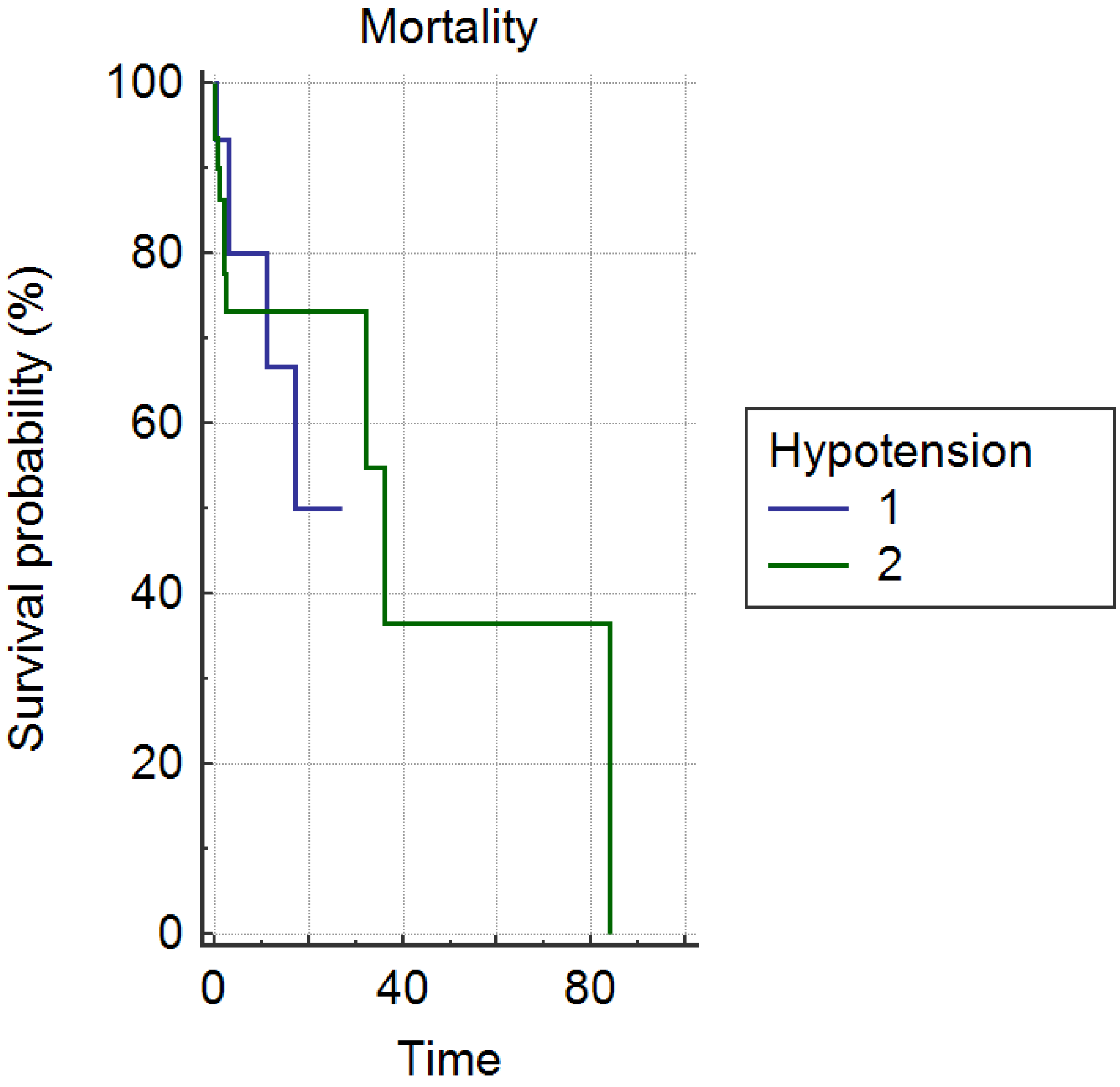

Supplementary Figure S1(f) Kaplan-Meier analysis showing there is no difference in survival rate between systemic capillary leak syndrome patients with death and those without according to the presence of leukocytosis (1: white blood cell counts > 15,000 and 2: white blood cell counts < 15,000/mm^3) (p=0.265).

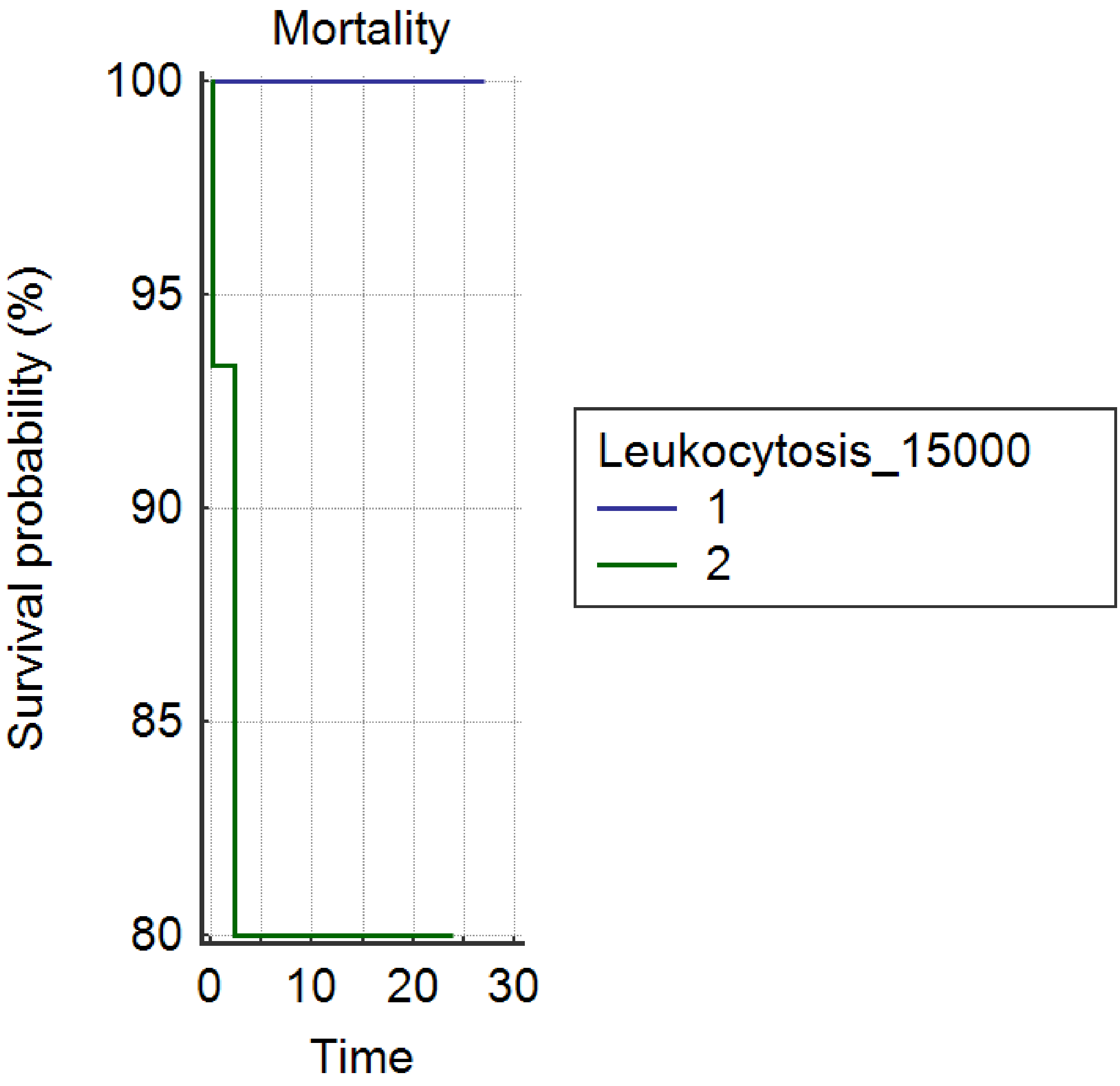

Supplementary Figure S1(g) Kaplan-Meier analysis showing there is no difference in survival rate between systemic capillary leak syndrome patients with death and those without according to the use of steroids (1: use of steroids and 2: no use of steroids) (p=0.705).

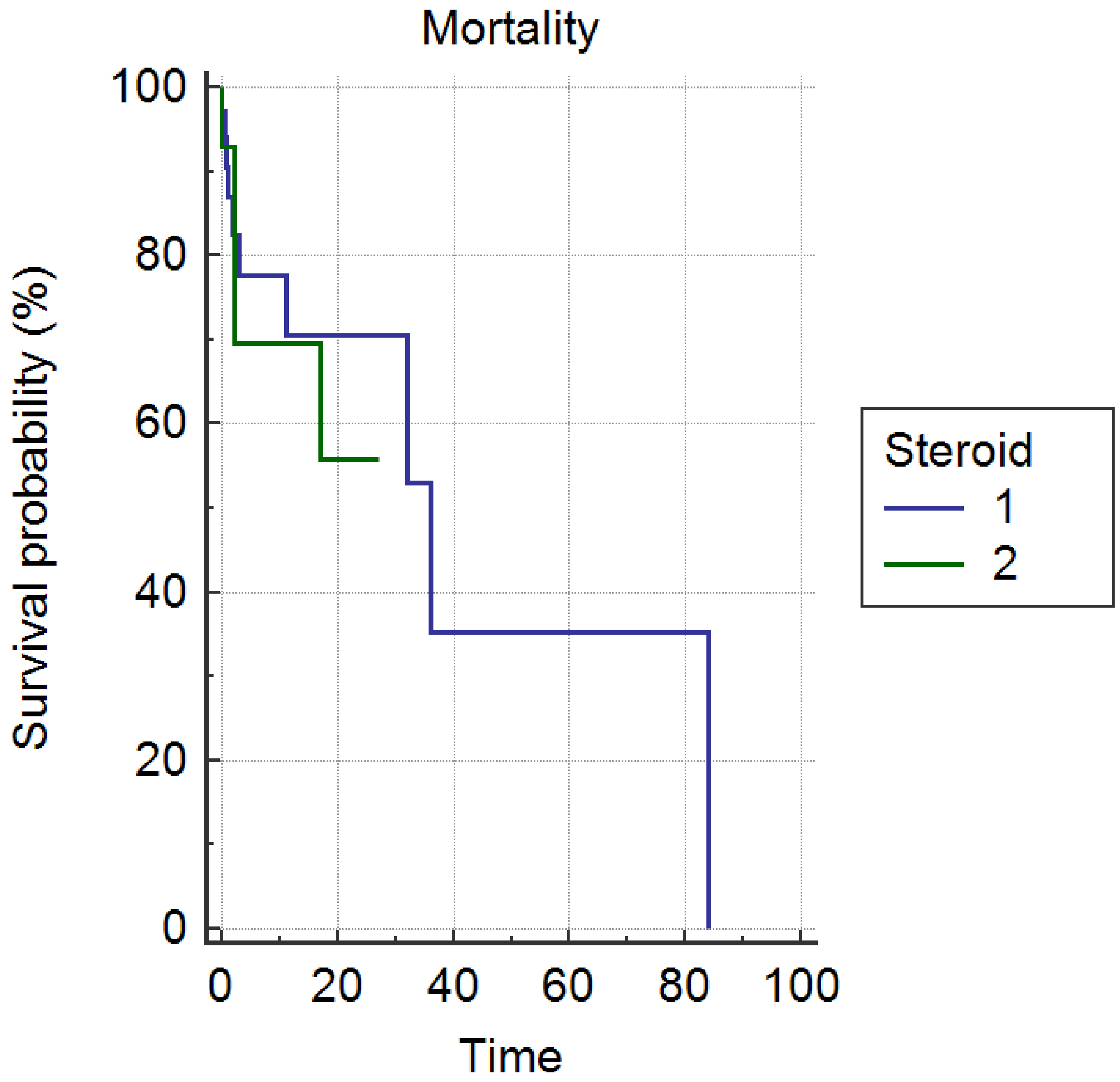

Supplement: Supplementary file 1 [file jcm-07-00418-s001.pdf]
